# Persistent hypoglossal arteries with aneurysmal dilation of left hypoglossal artery: a rare case report and review of the literature

**DOI:** 10.1259/bjrcr.20150301

**Published:** 2016-05-31

**Authors:** Shaileshkumar Garge, Vinu Moses, Shyamkumar Keshava, Munawwar Ahmed, Ranjit Moorthy

**Affiliations:** Department of Radiology and Neurosurgery, Christian Medical College, Vellore, India

## Abstract

Persistence of foetal anastomoses between carotid and vertebrobasilar arteries is a well-known anomaly, although bilateral persistence of these anastomoses is uncommon. Persistent hypoglossal artery is one of the four anastomotic vessels between the carotid and the vertebrobasilar arterial systems. Persistence of bilateral hypoglossal arteries with other associated anomalies is exceptionally uncommon and may result in unusual symptoms or have implications for therapy. We report an incidentally detected case of bilateral persistent hypoglossal arteries with an associated finding of hypoplastic vertebral arteries, describe their embryology and consider the potential clinical implications of this finding.

## Summary

The four types of persistent foetal anastomosis between the carotid and vertebrobasilar arteries are (from cranial to caudal) the persistent trigeminal, otic, hypoglossal and proatlantal arteries. Persistence of hypoglossal arteries is uncommon, with an incidence of 0.1–0.2%.^1^ To the best of our knowledge, only three case reports of bilateral persistent hypoglossal arteries have been reported in the literature; bilateral persistent hypoglossal arteries with associated fusiform aneurysmal dilatation of hypoglossal arteries have not been described before.^2–4^ We present the case of a patient with perimesencephalic subarachnoid haemorrhage (SAH) who had bilateral persistent hypoglossal arteries arising from the cervical segment of the internal carotid arteries that entered the skull *via* the hypoglossal canal and united with the lower portion of the basilar artery with fusiform aneurysmal dilatation on the left side towards the basilar segment along with hypoplastic bilateral vertebral arteries.

## Clinical presentation

A 60-year-old female patient presented with acute, severe headache, and giddiness and vomiting for 6 days. She was neither diabetic nor hypertensive. Neurological examination revealed no focal neurological deficit and a Glasgow Coma Scale score of 15/15.

## Differential diagnosis

As there was no history of trauma, cerebral aneurysmal rupture causing an SAH was suspected.

## Investigation/imaging findings

**Figure 1.**
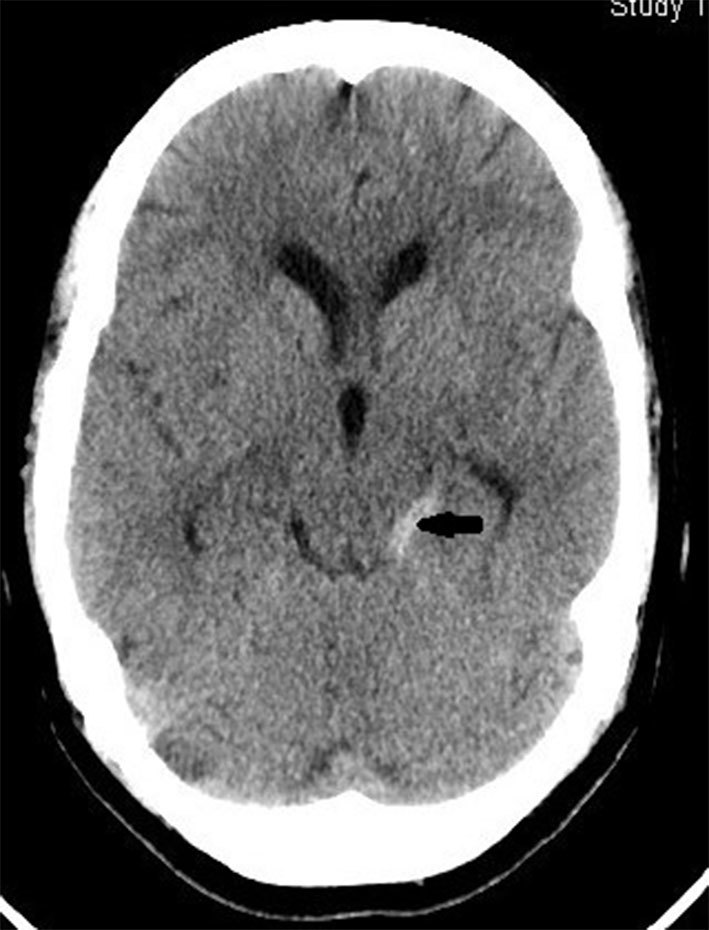
CT scan of the brain at the level of the mid-brain showing hyperdense blood in the perimesencephalic cistern on the left side (arrow).

**Figure 2.**
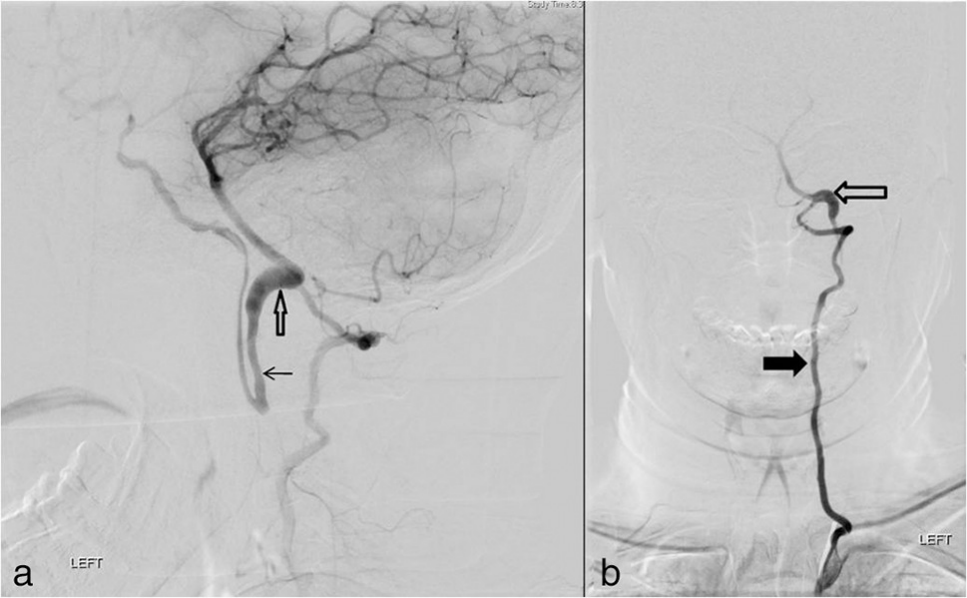
Left vertebral artery digital subtraction angiography, lateral projection (a) and Towne’s projection (b), showing the persistent hypoglossal artery that arose from the cervical segment of the internal carotid artery apparently followed the normal course of vertebral arteries and then took a slight dorsal course to enter the cranium *via* the hypoglossal canal before joining the proximal basilar artery (arrows) with an associated fusiform aneurysmal dilation of the segment towards the basilar artery (open arrows) and the mildly hypoplastic vertebral artery (thick arrow).

**Figure 3.**
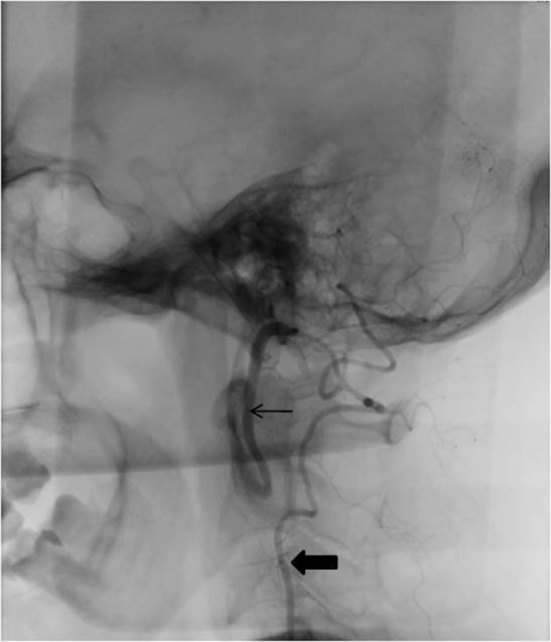
Right vertebral artery digital subtraction angiography, lateral projection (native image for bony landmarks) showing a hypoglossal artery similar to the left side but without any dilation (arrow). Hypoplastic vertebral artery is noted (thick arrow).

Axial section plain CT of the brain (120 kV, slice thickness 5 mm) at the level of the mid-brain showed perimesencephalic SAH (Figure 1, arrow). Digital subtraction angiography (DSA) showed “anomalous arteries arising from the cervical segment of the internal carotid arteries at the C2 vertebral level on both sides” (Figures 2a and 3, arrow) running posteriorly to join the proximal basilar artery, “fusiform aneurysmal dilation of the left hypoglossal artery towards its basilar segment” (Figure 2a,b, open arrow) and “mildly hypoplastic vertebral arteries on both sides” (Figures 2b and 3, thick arrows).

## Treatment

The patient was kept under observation and managed conservatively with analgesics and anticonvulsant.

## Outcome and follow-up

**Figure 4.**
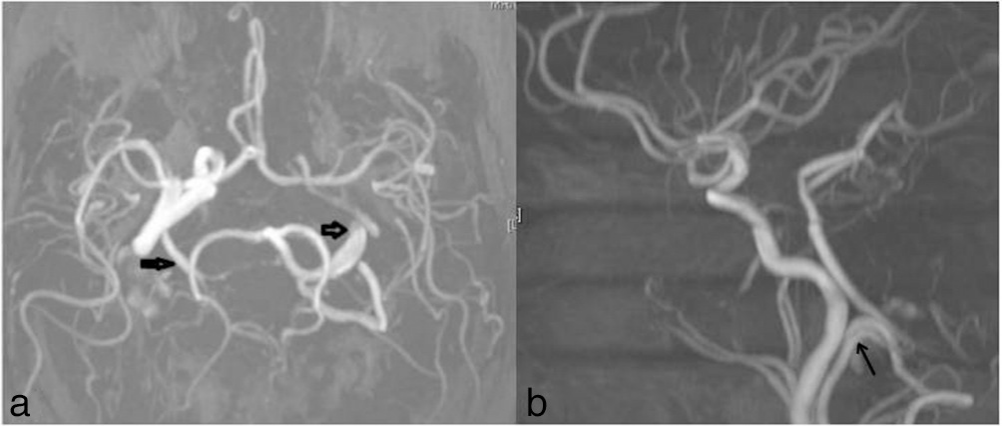
MR angiography maximum intensity projection images, axial (a) and sagittal (b), showing bilateral persistent hypoglossal arteries with aneurysmal dilation on the left hypoglossal artery (arrows).

The patient improved symptomatically and was discharged on the fourth day. Work-up was performed with an MRI and MR angiography of the brain at the 1-month follow-up visit, which reconfirmed the DSA findings of “bilateral persistent hypoglossal arteries with fusiform aneurysmal dilation of the left hypoglossal artery” (Figure 4, arrows). During this period, the patient remained asymptomatic.

## Discussion

At the 4–5-mm embryonic stage, the hindbrain is supplied by two parallel neural arteries. These two neural arteries supply blood from the carotid system *via* the trigeminal, otic, hypoglossal and proatlantal arteries.^5^ As the posterior communicating arteries develop, these anastomotic arteries start regressing.^6^ The lifespan of these arteries is about a week. By the 12-mm embryo stage, all the anastomoses between the carotid and vertebrobasilar arteries normally disappear.

Failure of this obliteration results in the persistence of embryonic arteries, which, to a variable extent, leads to hypoplasia of the vertebrobasilar system, as in our case of bilateral persistent hypoglossal arteries. Persistent hypoglossal artery is the second most common persistent carotid–basilar anastomosis (the most frequent is the trigeminal artery).^7^ Its incidence is reported to be 0.1–0.2%;^1^ the presence of bilateral persistent hypoglossal arteries is extremely uncommon. To the best of our knowledge, only three cases of bilateral persistent hypoglossal arteries have been reported.

Similar to the proatlantal artery, the persistent hypoglossal artery arises from the extracranial internal carotid artery. There are two important differentiating features between these two anastomoses: (1) the suboccipital horizontal course is characteristic of vertebral and proatlantal arteries, while the hypoglossal artery lacks this horizontal course. (2) The proatlantal artery enters the skull through the foramen magnum, whereas the hypoglossal artery enters the skull through the hypoglossal canal. Therefore, a small dorsal curve will be enough for the hypoglossal artery, but the proatlantal artery extends much more posteriorly and horizontally.^8^

While persistent hypoglossal artery is usually an incidental finding, it may be of clinical significance in some situations. Usually aneurysms develop at the bifurcations of arteries. In our case, a short segment fusiform aneurysmal dilatation was seen in the left persistent hypoglossal artery towards the basilar segment. Aneurysms in bilateral persistent hypoglossal arteries are yet to be reported.^9^

In our case, where the patient presented with perimesencephalic SAH, DSA showed bilateral persistent hypoglossal arteries with bilateral mildly hypoplastic vertebral arteries. Angiographically, no cause was identified for the SAH. Follow-up MRI and MR angiography of the brain also did not reveal any cause for the SAH. The aneurysmal dilatation of the persistent hypoglossal artery did not have any features of hypoglossal nerve compression on clinical examination.

Knowledge of variations in vascular anatomy is important, especially when dealing with a patient who has presented with a significant clinical problem such as acute SAH. In our case, as the aneurysmal dilation was located in the extradural segment, it was excluded as a possible cause of the SAH. When DSA is repeated after an initial negative study in patients with SAH, an aneurysm may be revealed in 2–24% of patients.^10^ It is reasonable to avoid repeat testing in patients who present with a clear perimesencephalic bleeding pattern on initial head CT and low clinical severity grade as long as the initial DSA was not a technically difficult study or complicated by vasospasm. Otherwise, repeat imaging with DSA with three-dimensional rotational angiography or multidetector CT angiography 1–2 weeks following the onset of perimesencephalic SAH is reasonable. A re-bleed suggests the presence of an occult aneurysm and is an indication for repeat DSA.^10^ As our case presented with acute perimesencephalic SAH, the cause of which could not be determined, and clinically improved with conservative management, the patient will be followed up as any other case of angiographically occult SAH.

## Learning points

1. Knowledge of variation in vascular anatomy is important, especially in patients with significant clinical problems such as acute SAH.
2. This case is unique and rare as bilateral persistent hypoglossal arteries with fusiform aneurysmal dilation were incidentally detected on the same side as the SAH, but as the location of the aneurysmal dilation was predominantly extradural, it was less likely the cause of the SAH.
3. When DSA is repeated after an initial negative study in patients with SAH, an aneurysm may be revealed in 2–24% of patients. It is reasonable to avoid repeat testing in patients who present with a clear perimesencephalic bleeding pattern on initial head CT and low clinical severity grade as long as the initial DSA was not a technically difficult study or complicated by vasospasm.

## Consent

Informed consent to publish this case (incl. images and data) was obtained and is held on record.
